# Assessment of Selected Surface and Electrochemical Properties of Boron and Strontium-Substituted Hydroxyapatites

**DOI:** 10.3390/molecules29030672

**Published:** 2024-01-31

**Authors:** Joanna Kolmas, Pavlo Samoilov, Aneta Jaguszewska, Ewa Skwarek

**Affiliations:** 1 Department of Pharmaceutical Chemistry and Biomaterials, Faculty of Pharmacy, Medical University of Warsaw, ul. Banacha 1, 02-097 Warsaw, Poland; joanna.kolmas@wum.edu.pl (J.K.);; 2 Department of Radiochemistry and Environmental Chemistry, Institute of Chemical Sciences, Faculty of Chemistry, Maria Curie-Skłodowska University, 3 Maria Curie-Skłodowska Sq., 20-031 Lublin, Poland; wasd0528@gmail.com

**Keywords:** pHpzc, pH_IEP_, zeta potential, surface charge, hydroxyapatite

## Abstract

Tissue engineering is an interdisciplinary field of science that has been developing very intensively over the last dozen or so years. New ways of treating damaged tissues and organs are constantly being sought. A variety of porous structures are currently being investigated to support cell adhesion, differentiation, and proliferation. The selection of an appropriate biomaterial on which a patient’s new tissue will develop is one of the key issues when designing a modern tissue scaffold and the associated treatment process. Among the numerous groups of biomaterials used to produce three-dimensional structures, hydroxyapatite (HA) deserves special attention. The aim of this paper was to discuss changes in the double electrical layer in hydroxyapatite with an incorporated boron and strontium/electrolyte solution interface. The adsorbents were prepared via dry and wet precipitation and low-temperature nitrogen adsorption and desorption methods. The specific surface area was characterized, and the surface charge density and zeta potential were discussed.

## 1. Introduction

Tissue engineering is an interdisciplinary field of science combining knowledge from biology, chemistry, clinical medicine, and technical sciences, as well as materials and biomedical engineering. Its main goal is to regenerate damaged tissues and internal organs and to create new tissues in the case of completely removed structures. There are three key elements in tissue engineering: biological cells, a structural matrix enabling cell colonization, and growth factors [1]. Great hopes have been placed in the resolution of tissue defects using tissue engineering. Tissue engineering is a relatively new solution in regenerative medicine, and aims to restore the structures and functions of damaged body structures [2]. Tissue engineering involves the population of patient stem cells into a manufactured scaffold, followed by implanting the entire structure in place of diseased or removed tissue. A biomaterial is a biocompatible material that can be subject to long-term contact with living tissues and body fluids without a negative reaction from the body [3]. The purpose of these biomaterials is to partially or completely replace a tissue or organ in the body and take over its functions. The necessary properties of biomaterials used in materials engineering include, among others, biocompatibility, bioactivity, biofunctionality, lack of toxic reactions, lack of allergic reactions, appropriate mechanical and fatigue strength adapted to the required application, and biodegradability or bioresorbability [3]. Bioactive ceramics are an important group of materials in tissue engineering. Ceramic materials easily form a chemical bond with tissue. Calcium phosphates (CaP) are characterized by excellent biocompatibility due to their chemical similarity to bones [4]. They have osteoconductive properties, i.e., they support the process of creating new bone tissue. Calcium phosphates can crystallize into various salts such as hydroxyapatite (HA) or tricalcium phosphate (TCP), depending on the Ca:P ratio. Hydroxyapatite (HA) is chemically similar to the mineral component of bones and mineralized tissues in mammals. It also naturally occurs in the form of the mineral Ca_10_(PO_4_)_6_(OH)_2_, and the ideal Ca:P ratio is 10:6. Among tricalcium phosphates (TCP), there are high-temperature (α-TCP) and low-temperature (β-TCP) varieties. Despite the very good biological properties of HA and TCP, their use is limited. This is due to slow degradation (in the case of HA) and particularly low mechanical strength (lower than natural bone). The dissolution rate of these materials decreases in the following order: amorphous calcium phosphate » α—TCP » β—TCP » HA. The biological and mechanical properties of synthetic calcium phosphates vary with changes in their degree of crystallinity, grain size, porosity, and chemical composition [5]. Deterioration of mechanical properties is noted with an increase in the share of the amorphous phase, microporosity, and grain size [6].

Among other inorganic calcium phosphates, hydroxyapatite with the formula Ca_10_(PO_4_)_6_(OH)_2_ is one of the most common minerals used in various fields, in particular in medicine and pharmacy [7]. It owes its popularity to its significant similarities with bone and hard dental tissue and their mineral fractions, which contain nanocrystalline carbonated hydroxyapatite deficient in calcium and hydroxyl groups. For decades, due to its high biocompatibility, non-toxicity in human tissues, and non-immunogenicity, as well as high affinity for natural polymers and osteogenic potential, HA has been used as a bone substitute material, a coating for metallic implants to ensure osseointegration, and as a component of bone composite biomaterials. HA shows high susceptibility to ionic substitutions; therefore, it is possible to introduce various ions into its structure, affecting its physicochemical, biological, and mechanical properties. For example, the partial substitution of calcium with silver cations grants hydroxyapatite the capacity for additional antibacterial activity [8,9]. Additionally, the introduction of carbonate ions in place of orthophosphates causes a significant reduction in crystal sizes and increases solubility, while the substitution of OH groups with fluoride ions leads to an increase in the hardness of apatite [8,9,10]. Strontium (Sr) is a very promising element for substituting calcium cations in HA. Sr has a two-way effect on bone tissue. It positively affects the differentiation and proliferation of osteoblasts and the synthesis of bone matrix proteins occurring in these cells. Thus, it exhibits a bone-forming effect [11,12]. On the other hand, it inhibits formation and differentiation of osteoclasts and promotes their apoptosis, which weakens the process of bone resorption. Strontium in the form of strontium ranelate, thanks to the above mechanisms of action, is accepted in many countries as a drug for the treatment of osteoporosis. However, long-term systemic administration of strontium salt preparations results in many severe side effects [11]. Hence, research is being carried out on the possibility of intraosseous introduction of strontium ions together with bone substitutes that fill tissue defects. Hydroxyapatite is one such substitute that has attracted considerable interest [12,13]. Regardless of the Sr concentration in hydroxyapatite, no signs of cytotoxicity have been reported [14,15]. In vivo tests in animal models have shown that in the case of scaffolds based on Sr-HA, larger volumes of newly formed bone were observed compared to pure HA. In addition, Sr substitution improves the mechanical properties of hydroxyapatite [16].

Boron is a trace element that plays a very important role in many life processes, especially in the metabolism of calcium and the proper development of bone tissue. Many studies indicate that boron has a beneficial effect on the regeneration of osseous tissue [17]. It is able to promote the proliferation and differentiation of osteoblasts. Studies carried out on preosteoblast cells have also shown the beneficial effect boron has on mineralization processes, expression of the gene that encodes type I collagen, and increasing sialoprotein and osteocalcin content in preosteoblast cells [18]. Previous research on the introduction of borate ions into the structure of hydroxyapatite indicated that it is not as easy as in the case of integration of strontium. Ternane et al., during synthesis in a solid state, set the limit for the introduction of borate ions at x = 0.5 mol per mol of HA [19]. Above this value, other crystalline phases and CaO were formed. Moreover, spectroscopic analysis showed the presence of not only BO_3_^3−^ (orthoborates), but also BO_2_^−^ (metaborates). Barheine et al. confirmed these results [20]. At the same time, a schematic model of the structure of hydroxyapatite enriched with boron ions was proposed, where borates would be located in the disordered surface layer surrounding the crystalline apatite core [20].

In the available literature, only a few studies have been devoted to the possibility of introducing borate ions into the hydroxyapatite structure, and even less to the co-introduction of boron and strontium [19,20,21,22,23]. Electrochemical studies of materials such as various types of Sr- or B-substituted hydroxyapatite are very important in terms of examining their biodegradability, bioresorbability, and biofunctionality. Preliminary studies have indicated great biocompatibility of such hydroxyapatites [23]. In order to continue research on apatite enriched with Sr and/or B, we decided to investigate the surface and electrochemical properties of these materials.

## 2. Results and Discussion

**Figure 1:**
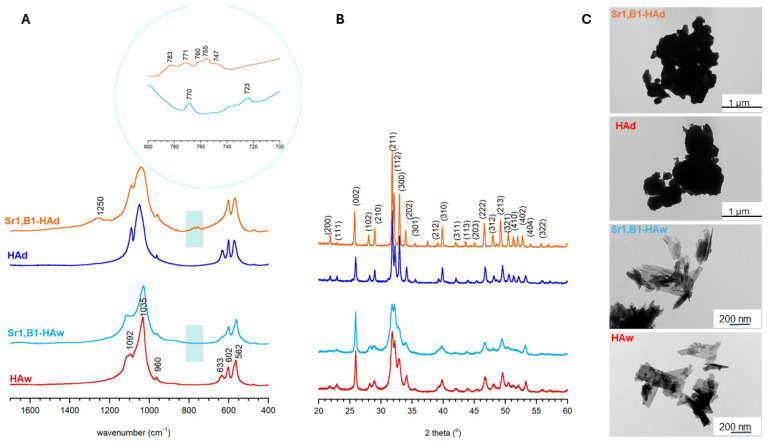
Representative FT-IR spectra (**A**), PXRD diffractograms (**B**), and TEM images (**C**).

Acquired FT-IR spectra and PXRD diffractograms confirmed the successful synthesis of hydroxyapatite samples using both wet and dry methods (see Figure 1). Similar to our prior investigations, samples produced through the wet method exhibited a nanocrystalline structure, as indicated by the broad reflections in the PXRD pattern, and broadened, indistinct phosphate bands in the FT-IR spectra [23]. Conversely, the spectra of samples synthesized using the solid-state method (dry method) revealed a microcrystalline nature. PXRD patterns illustrated the presence of a homogeneous apatitic phase in the powders, without any discernible crystalline impurities.

Table 1 and Table 2 present the content of strontium and boron in the materials. It is noteworthy that, in accordance with previous research, the incorporation of foreign ions (B and Sr) into the hydroxyapatite structure was more effective using dry synthesis, and this was particularly evident in the case of borate ion substitution [23]. An analysis of the unit cell parameters (a and c) of the examined powders was warranted. In the case of strontium ion substitution, the cell expanded along both the a and c axes, consistent with earlier studies. The introduction of borate ions did not significantly alter the cell parameters of samples obtained through the wet method, likely due to the limited amount of boron introduced. However, using the dry method, an increase in the c parameter was observable, aligning with previous reports [18,19,23].

This publication presents TEM photos (Figure 1) and the size of the specific surface, determined using the BET and Langumir isotherm (Table 1 and Table 2), for selected samples, namely Sr1, B1+HAd, HAd, Sr1, B1+Haw, and HAw. These exhibits show that while materials obtained using the wet method had a smaller overall particle size of approximately 200 nm, they had a larger specific surface, which is an expected correlation because smaller particles have a more developed surface and more micro and mesopores. The opposite situation was observed for samples obtained through dry sintering. The samples combined with each other and created a less developed structure of the specific surface. Samples obtained differently, through wet versus dry methods, differ in surface activity and adsorption capacity depending on the specific surface area. Therefore, measurement of specific surface area is important for determining the activity and adsorption capacity of materials.

Table 1 and Table 2 show the selected structural parameters of hydroxyapatite (HA) and its admixtures of boron and strontium obtained through the wet method (Table 1) and the dry method (Table 2). It can be seen that the specific surface area and the structural parameters of the tested compounds obtained via the wet method were much larger than those obtained via the dry method, which indicates that the synthesis method, especially the temperatures involved, had a large and present effect. It can be concluded that adsorbents with a larger specific surface area possess better adsorption properties. In the adsorbents obtained via the wet method, the average pore radius was larger. Such a large pore size, as observed in these samples, represents a surface structure conducive to their application as a biomaterial in living organisms. The specific surface area of hydroxyapatite (HAw) as obtained by the wet method was smaller than that of the adsorbents containing the admixtures of strontium and boron. It was found that the larger the content of boron and strontium, the larger the surface area, which may be due to the substitution of calcium ion types in hydroxyapatite with boron and strontium ions. Moreover, the specific surface area of hydroxyapatite (HAd) obtained via the dry method was larger than that of the adsorbents containing admixtures of strontium and boron. The adsorbent with the largest content of boron and strontium had a surface area half that of the HAd. This may be due to the higher temperature involved in the synthesis method. The samples obtained via the wet method have a much better developed entrustment, as evidenced by the total pore volume, among other observations.

Table 1 and Table 2 show a clear impact of the presence of strontium and boron ions on the surface properties of the tested samples. This is as a result of the exchange of metal ions with Ca ions in the hydroxyapatite crystal lattice; the radius of the exchanged cations does not differ more than 15% from that of Ca^2+^, and their electronegativity is high. The ion exchange does not take place on the hydroxyapatite surface, but probably in the natural CaII and CaI channels in the structure of hydroxyapatite; boron or strontium adsorption can occur on the surface of phosphate or hydroxyl groups with the release of one or two protons by the adsorption of one or two surface groups of the tested samples, respectively.

**Figure 2:**
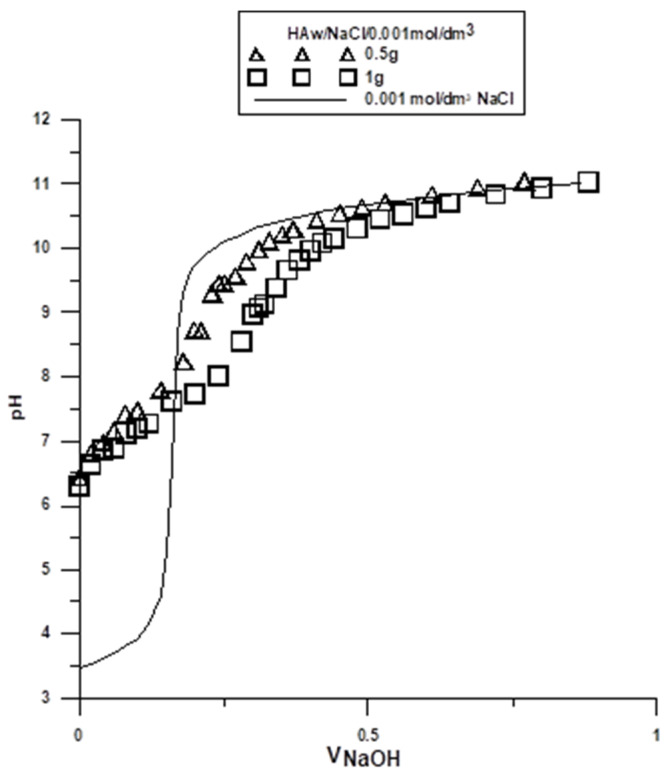
Curves of the potentiometric titration of hydroxyapatite (HA) obtained via the wet method.

**Figure 3:**
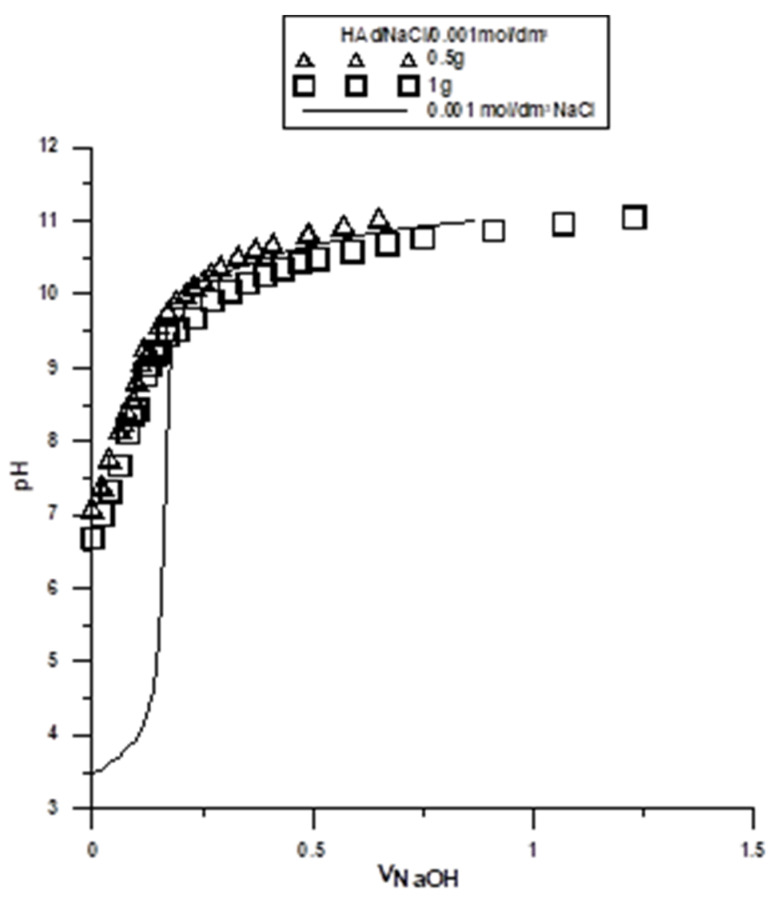
Curves of the potentiometric titration of hydroxyapatite (HA) obtained via the dry method.

The potentiometric titration of suspensions is the most commonly used method for determination of surface charge and the position of the point of zero charge (pH_PZC_) in the solid/electrolyte solution system. These measurements, together with the zeta potential, are helpful in determining the structure of the electrical double layer (EDL) at the interface. The point where the surface concentration of the positively charged groups is equal to the concentration of the negatively charged groups is called the point of zero charge. In order to determine the pH_PZC_, two hydroxyapatite weights were titrated: 0.5 g and 1 g, both in NaCl 0.001 mol/dm^3^. Titration curves for each of the sample weights were collected on one graph (Figure 2 and Figure 3). The intercept of the curves corresponds to the pH value at which pH_PZC_ occurs. Similar titrations were performed for all tested samples, and the results are presented in Table 3 and Table 4.

Based on the results presented in Table 3 and Table 4, the samples obtained via the wet method had lower pH_PZC_ values than those obtained via the dry method. In the tested pH range, a smaller positively charged surface was observed in the samples obtained via the wet method, and in the case of the dry samples, a larger positively charged surface was observed. This phenomenon represents an evident effect of the synthesis method.

The samples obtained via the dry method may collect ions that are not fully coordinated on the sample surface, which leads to the occurrence of local charges. In contrast, exposure of such a surface to water for the samples obtained via the wet method leads to the formation of surface hydroxyl groups. These groups are able to dissociate hydrogen ions. Hydrogen ions that are released from the surface hydroxyl groups of the tested samples move away from the solid/electrolyte solution interface towards the depth of the solution. This process continues until there is an equilibrium established between the surface and the solution. The surface charge at the solid/water electrolyte interface can be formed by selective adsorption of ions, dissociation of surface -OH groups, adsorption of carrier electrolyte ions on these -OH groups, isomorphic substitution, or the accumulation of electrons.

For the samples obtained via the wet method, an evident effect of the synthesis conditions on the density of the surface charge of hydroxyl groups was observed. The results in Table 3 show that the greater the boron content of the sample, the smaller the pH_PZC_ value. In the case of the samples in Table 4, it is shown that the incorporation of ion admixtures increased the pH_PZC_.

The pH_PZC_ parameters for the individual samples obtained via both the wet and dry methods assist in explaining the adsorption processes taking place on the surfaces of these samples when they are applied as biomaterials in living organisms.

One of the most important parameters characterizing the electrical double layer is the zeta potential. The zeta electrokinetic potential is the measured potential in the slip plane relative to the point in the volume of the solution. It is determined using the Smoluchowski equation:

ζ = αu/DF η

where:

η—the viscosity,
D—the dielectric constant,
F—the Faraday constant, and
α—the coefficient, depending on the shape of the particle (for spherical particles α = 6π, and for cylindrical particles α = 4π).

**Figure 4:**
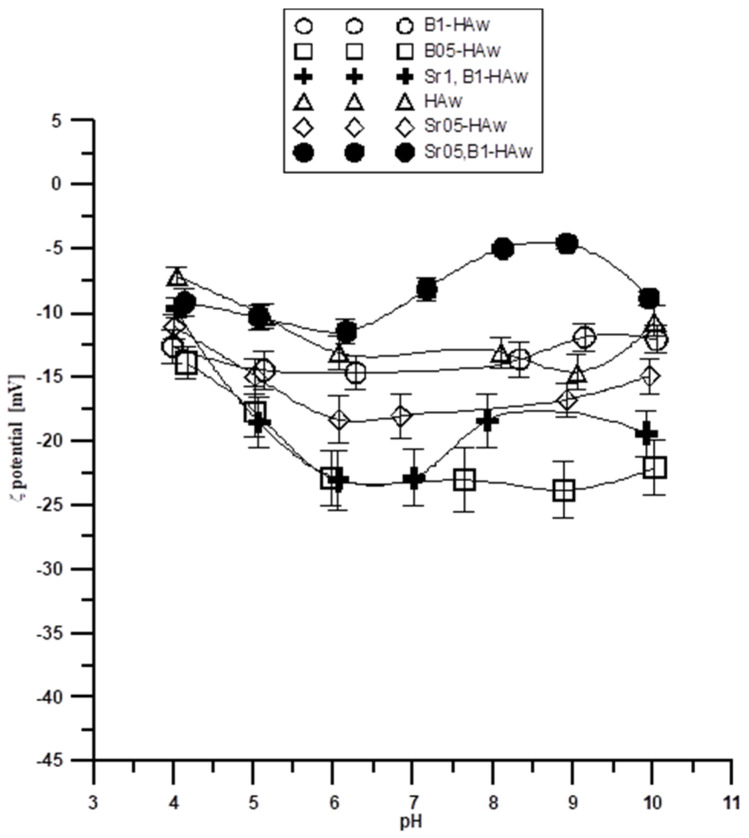
Dependence of the zeta potential on pH for the samples obtained via the wet method.

**Figure 5:**
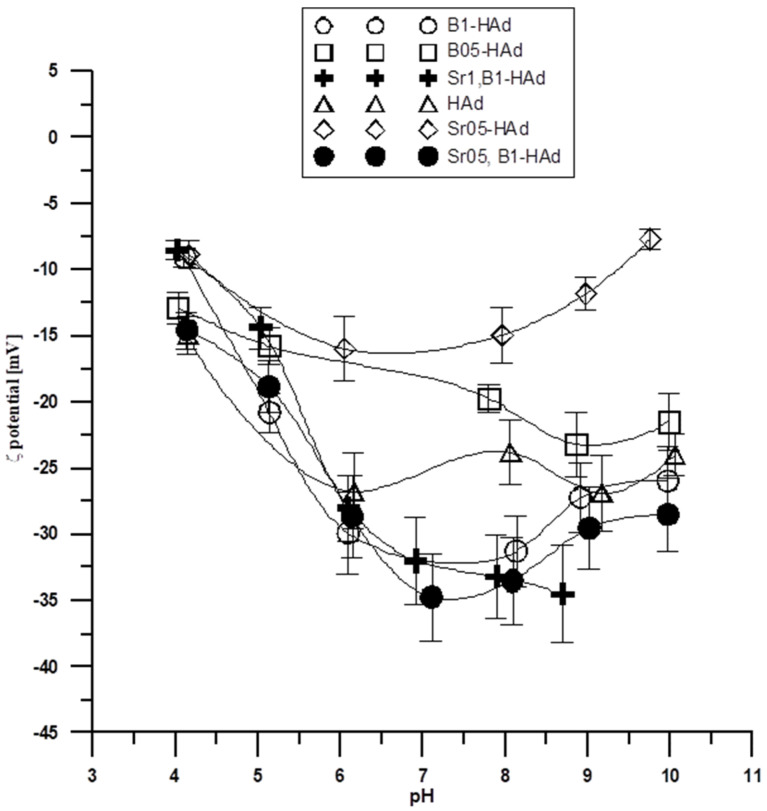
Dependence of the zeta potential on pH for the samples obtained via the dry method.

Another important parameter characterizing the double electrical layer is the isoelectric or pH_IEP_ point. The zeta potential is zero at this point; the surface concentration of positively charged groups is equal to the surface concentration of negatively charged groups. As follows, from Figure 4 and Figure 5, the potential relationship ζ indicates that the zeta potential changes with increasing pH. The dependence of the zeta potential as a function of pH suggests that the pH_IEP_ is less than 4 for all samples from both the wet and dry methods. Small pH_IEP_ values can indicate that the diffusion layer is mainly negatively charged in the entire tested pH range. For the porous adsorbents, according to the nitrogen adsorption and desorption (ASAP) tests, the energy heterogeneity of the surface is characteristic. Thus, there is a possibility that for the tested electrolyte concentration, double layers from the individual pore walls can overlap, so the pores may be “clogged” during electrophoresis and the properties of this part of the solid surface are not revealed. This is due to the fact that a significant part of the charge can be compensated for inside the pores of the particle, and only the part that comes from the ionized groups on the surface of the adsorbent is responsible for electrophoretic mobility.

The electrokinetic behavior of the particles in the colloidal system is important in determining their stability and rheological behavior in the tested systems. Therefore, the zeta potential plays an important role in determining the stability of the samples in Table 1 and Table 2, which are usually susceptible to aggregation. The stability and rheological properties of suspensions are important in the production of technologically advanced substances, e.g., pharmaceuticals.

Stability, i.e., the ability of particles to remain in the form of a colloidal dispersion, is a very important quality. The processes of coagulation and sedimentation result in delamination of the sample. The zeta potential can be used to quantify dispersion stability. It is assumed that the dispersion is stable when the absolute value of the zeta potential is >30 mV. The zeta potentials for the tested samples in the tested pH range from three to 11. The electrolyte concentration of 0.001 mol/dm^3^ is in the range of −5 mV to −25 mV for the samples obtained using the wet method, and in the range of −5 mV to −35 mV for the samples obtained using the dry method. This suggests that in most of the tested ranges, the selected systems are colloidally unstable and can delaminate. This effect may be due to particle aggregation as a result of a decrease in the absolute values of the zeta potential. Comparison of the results for pure hydroxyapatite and its admixtures of strontium and boron shows that at the same electrolyte concentration, pure hydroxyapatite assumes different values, and thus the clear influence of the presence of strontium and boron ions for both types of samples obtained (dry and wet) can be seen. The presence of Sr and B ions can compensate for the negative charge and also introduce an additional positive charge to the compact part of the electrical double layer, which results in differences in the zeta potential for individual samples.

## 3. Materials and Methods

The synthesis of hydroxyapatites containing borates, strontium, or both was achieved via two different methods: a standard precipitation (wet) method in an aqueous environment, and a dry method using high temperature. These are described in detail in [23].

Briefly, the wet method used calcium nitrate tetrahydrate Ca(NO_3_)_2_∙4H_2_O, diammonium hydrogen phosphate (NH_4_)_2_HPO_4_, orthoboric acid H_3_BO_3_, and strontium nitrate Sr(NO_3_)_2_ as sources of calcium, phosphorus, boron, and strontium, respectively. In the dry method, the sources of calcium, phosphorus, boron, and strontium were calcium carbonate CaCO_3_, diammonium hydrogen phosphate (NH_4_)_2_HPO_4_, boric acid H_3_BO_3_, and strontium carbonate SrCO_3_, respectively. All reagents were weighed so that the molar ratio (Ca + Sr)/(B + P) was equal to 1.67. In the wet method, a solution containing calcium ions (and strontium ions) was added dropwise to a solution containing phosphate ions (and orthoborate ions) with constant stirring. Upon completion of the dropwise addition, the pH of the resulting slurry was adjusted to 11.0 with aqueous ammonia. Stirring was continued for another 2 h at 70 °C. After this time, the solution was left at room temperature for 24 h for aging. Then, the resulting white precipitate was filtered off and washed several times with distilled water to remove ammonia and soluble synthesis products. The precipitate was dried for 24 h at 120 °C. 

In the dry synthesis process, the weighed reagents were mixed together in a ball mill for 30 min. The resulting tablets were compressed in a hydraulic press using a pressure of 5 tons/cm^2^. The pellets were placed in an oven and then calcined using the following temperature program: 450 °C for 12 h, 700 °C for 12h, and 1000 °C for 24 h. After this time, the tablets were crushed in a mortar and finely powdered. Finally, twelve different powders were obtained (see Table 5).

Analysis of strontium and boron content was performed via the ICP-OES method, which confirmed the synthesis of the assumed materials, and the PXRD patterns and FT-IR spectra were also determined for the samples.

PXRD experiments were carried out on a Bruker D8 Discover diffractometer (Billerica, MA, USA) using Cu Kα radiation (λ = 1.54 Å). The two theta range from 20° to 60° was utilized. The obtained diffractograms were used to obtain the unit cell parameters, which were processed by TOPAS-Academic V7 software. To analyze the chemical structure of the samples, FT-IR spectroscopy was used in transmission (KBR pellets) mode. A PerkinElmer Spectrum 1000 spectrometer (Waltham, MA, USA) was used to obtain the spectra in the 4000–400 cm^−1^ range, with a spectral resolution of 2 cm^−1^, and 30 scans. For the measurement of boron and strontium content, the ICP-OES method was used (Perkin Elmer Optima 3100XL). Before analysis, the samples were dissolved in concentrated (63%) ultrapure nitric acid. The morphology of the powders was analyzed via transmission electron microscopy (TEM, Jeol JEM1400, JEOL, Kishima, Japan). To perform the measurements, a drop of sample suspended in pure ethanol was placed on a Cu/formvar grid, dried, and then analyzed under an accelerating voltage of 80 kV.

The research was carried out using an ASAP (Accelerated Surface Area and Porosimetry System), potentiometric titration, and measurements of electrophoretic mobility to determine the zeta potential.

Using the results from nitrogen adsorption and desorption, the porosity and specific surface area of the synthesized samples were determined. The nitrogen adsorption/desorption isotherms at 77 K were measured using the ASAP 2405 sorption analyzer from Micromeritics Instruments, Co., Norcross, GA, USA. The specific surface area of the HA sample was calculated using the BET method and porosity was determined using the Barrett, Joyner, and Halenda (BJH) procedure.

The zeta potential was measured using the Zetasizer Nano-ZS (Malvern, Almelo, The Netherlands). In the zeta potential calculations, the Smoluchowsky equation was also applied due to the κa of ~150. A NaCl solution with a concentration of 0.001 mol/dm^3^ was prepared. Then, the prepared solution was added to a 250 cm^3^ beaker containing 0.02 g of the previously weighed solid sample. The obtained system was sonicated using the Misonix Sonicator XL2020 ultrasonic probe (SpectraLab Scientific Inc., Markham, ON, Canada) for 3 min, and then the pH values were set to 11, 10, 9, 8, 7, 6, 5, and 4.

pH_PZC_ was also determined for the tested systems at the sample/0.001 mol/dm^3^ NaCl interface with two different adsorbent weights, 0.5 g and 1 g, due to the solubility of the samples. This was performed using a glass electrode (indicator), pHG201-8, and a calomel electrode (reference), REF 451, applying the system for potentiometric tests. The titrant (NaOH solution) was dosed using an automatic burette, Dosimat 665 by Metrohm. The instruments were connected to a computer which, using the titr_v3 program (written for the needs of our research at the Department of Radiochemistry and Environmental Chemistry), collected data from the pH-meter and the burette and controlled the burette based on the pH changes recorded by the pH-meter. Each time, 50 cm^3^ of electrolyte and 0.2 cm^3^ of HCl were poured into the measuring cell in order to reduce the pH of the starting solution.

## 4. Conclusions

- The acquired FT-IR spectra and PXRD diffractograms confirmed the successful syn-thesis of hydroxyapatite samples using both the wet and dry methods.
- The incorporation of foreign ions (B and Sr) into the hydroxyapatite structure is more effective using dry as opposed to wet synthesis.
- This paper presents the results of analyses using the adsorption method, and nitrogen desorption, potentiometric titration, and zeta potential measurements can be summarized as follows:
  o The specific surface areas of tested compounds obtained via the wet method are much larger than those obtained using the dry method, i.e., the influence of the synthesis method, especially the involved temperature, as well as the difference in strontium and boron ion content are evident.
  o The samples obtained with the wet method have smaller pH_PZC_ values than those obtained with the dry method in the tested pH range.
  o The zeta potential for the tested samples in the tested pH ranges (from three to 11) and the electrolyte concentration of 0.001 mol/dm^3^ is in the range of −5 mV to −25 mV for the samples obtained with the wet method, and in the range of −5 mV to −35 mV for those obtained using the dry method, i.e., in most of the studied ranges, the selected systems are colloidally unstable. The pH_IEP_ values for the individual samples are smaller than four.

## Figures and Tables

**Table 1:** Selected structural parameters of hydroxyapatite (HA) and its boron and strontium admixtures obtained via the wet method.

Parameters	HAw	Sr05-HAw	B05-HAw	B1-HAw	Sr05,B1-HAw	Sr1,B1-HAw
Strontium content [mol]	-	0.38 ± 0.01	-	-	0.33 ± 0.02	0.56 ± 0.02
Boron content [mol]	-	-	0.21 ± 0.03	0.35 ± 0.02	0.26 ± 0.02	0.24 ± 0.02
a unit cell parameter [nm]	0.9429	0.9436	0.9432	0.9429	0.9444	0.9451
c unit cell parameter [nm]	0.6876	0.6890	0.6880	0.6876	0.6890	0.6895
Surface area based on BET [m^2^/g]	62	96	109	72	120	142
Surface area based on Langmuir isotherm [m^2^/g]	85	140	159	100	175	207
Total pore volume from adsorption 1.7 nm < d < 300 nm by the BJH (Barrett–Joyner–Halenda) method [cm^3^/g]	0.31	0.50	0.59	0.34	0.65	0.68
Total pore volume from desorption 1.7 nm < d < 300 nm by the BJH (Barrett–Joyner–Halenda) method [cm^3^/g]	0.32	0.50	0.60	0.35	0.66	0.68
Average pores radius from BET method [nm]	20.55	20.87	21.92	19.33	21.90	19.17
Average pores radius from adsorption, BJH method [nm]	22.66	22.02	23.58	23.09	23.55	19.41
Average pores radius from desorption, BJH method [nm]	20.53	19.76	20.85	21.82	21.06	17.06

**Table 2:** Selected structural parameters of hydroxyapatite (HA) as well as its boron and strontium admixtures obtained via the dry method.

Parameters	HAd	Sr05-HAd	B05-HAd	B1-HAd	Sr05, B1-HAd	Sr1, B1-HAd
Strontium content [mol]	-	0.44 ± 0.03	-	-	0.40 ± 0.01	0.86 ± 0.02
Boron content [mol]	-	-	0.46 ± 0.02	0.90 ± 0.02	0.89 ± 0.03	0.79 ± 0.02
a unit cell parameter [nm]	0.9426	0.9412	0.9415	0.9409	0.9444	0.9448
c unit cell parameter [nm]	0.6886	0.6907	0.6883	0.6904	0.6915	0.6948
Surface area based on Langmuir isotherm [m^2^/g]	7	3	2	4	2	3
Total pore volume from adsorption 1.7 nm < d < 300 nm by the BJH (Barrett–Joyner–Halenda) method [cm^3^/g]	0.025	0.009	0.004	0.011	0.004	0.008
Total pore volume from desorption 1.7 nm < d < 300 nm by the BJH (Barrett–Joyner–Halenda) method [cm^3^/g]	0.025	0.008	0.004	0.012	0.004	0.008
Average pores radius from BET method [nm]	18.08	16.77	10.09	14.64	10.51	14.07
Average pores radius from adsorption, BJH method [nm]	27.34	35.72	23.99	19.53	36.91	20.79
Average pores radius from desorption, BJH method [nm]	24.10	30.65	29.18	15.06	31.20	16.16

**Table 3:** pH_PZC_ values for individual samples obtained via the wet method.

Samples	pHpzc
HAw	8.05
Sr05-HAw	8.44
B05-HAw	8.1
B1-HAw	6.79
Sr05, B1-HAw	8.77
Sr1, B1-HAw	8.22

**Table 4:** pH_PZC_ values for individual samples obtained via the dry method.

Samples	pHpzc
HAd	9.76
Sr05-HAd	>11
B05-HAd	>11
B-HAd	>11
Sr05,B1-HAd	10.67
Sr1, B1-HAd	>11

**Table 5:** Description of the obtained powders (n_B_ and n_Sr_—nominal value of boron and strontium added [mol]).

Wet Method	Dry Method	Nominal Composition	Description
HAw	HAd	Ca_10_(PO_4_)_6_(OH)_2_	Pure, unsubstituted hydroxyapatite
B1-HAw	B1-HAd	Ca_10_(PO_4_)_5_(BO_3_)(OH)_2_	Hydroxyapatite substituted with borate ions n_B_ = 1
B05-HAw	B05-HAd	Ca_10_(PO_4_)_5.5_(BO_3_)_0.5_(OH)_2_	Hydroxyapatite substituted with borate ions n_B_ = 0.5
Sr05-HAw	Sr05-HAd	Ca_9.5_Sr_0.5_(PO_4_)_6_(OH)_2_	Hydroxyapatite substituted with strontium ions n_Sr_ = 0.5
Sr1,B1-HAw	Sr1,B1-HAd	Ca_9_Sr(PO_4_)_5_(BO_3_)(OH)_2_	Hydroxyapatite substituted with strontium ions n_Sr_ = 1 and borate n_B_ = 1
Sr05,B1-HAw	Sr05,B1-HAd	Ca_9.5_Sr0.5(PO_4_)_5_(BO_3_)(OH)_2_	Hydroxyapatite substituted with strontium ions n_Sr_ = 0.5 and borate n_B_ =1

## Data Availability

Data are contained within the article.
